# Symptom clusters and unplanned hospital readmission in Chinese patients with acute myocardial infarction on admission

**DOI:** 10.3389/fcvm.2024.1388648

**Published:** 2024-05-20

**Authors:** Yijun Mao, Yuqiong Shi, Wenfang Qiao, Zhuo Zhang, Wei Yang, Haili Liu, Erqing Li, Hui Fan, Qiang Liu

**Affiliations:** ^1^ Department of Cardiology, Xianyang Central Hospital, Shaanxi, China; ^2^ Department of Nursing, Xianyang Central Hospital, Shaanxi, China; ^3^ Department of Orthopedic, Xianyang Central Hospital, Shaanxi, China

**Keywords:** acute myocardial infarction, symptom cluster, cross-section studies, latent class analysis, unplanned hospital readmission

## Abstract

**Backgroud:**

Acute myocardial infarction (AMI) has a high morbidity rate, high mortality rate, high readmission rate, high health care costs, and a high symptomatic, psychological, and economic burden on patients. Patients with AMI usually present with multiple symptoms simultaneously, which are manifested as symptom clusters. Symptom clusters have a profound impact on the quality of survival and clinical outcomes of AMI patients.

**Objective:**

The purpose of this study was to analyze unplanned hospital readmissions among cluster groups within a 1-year follow-up period, as well as to identify clusters of acute symptoms and the characteristics associated with them that appeared in patients with AMI.

**Methods:**

Between October 2021 and October 2022, 261 AMI patients in China were individually questioned for symptoms using a structured questionnaire. Mplus 8.3 software was used to conduct latent class analysis in order to find symptom clusters. Univariate analysis is used to examine characteristics associated with each cluster, and multinomial logistic regression is used to analyze a cluster membership as an independent predictor of hospital readmission after 1-year.

**Results:**

Three unique clusters were found among the 11 acute symptoms: the typical chest symptom cluster (64.4%), the multiple symptom cluster (29.5%), and the atypical symptom cluster (6.1%). The cluster of atypical symptoms was more likely to have anemia and the worse values of Killip class compared with other clusters. The results of multiple logistic regression indicated that, in comparison to the typical chest cluster, the atypical symptom cluster substantially predicted a greater probability of 1-year hospital readmission (odd ratio 8.303, 95% confidence interval 2.550–27.031, *P* < 0.001).

**Conclusion:**

Out of the 11 acute symptoms, we have found three clusters: the typical chest symptom, multiple symptom, and atypical symptom clusters. Compared to patients in the other two clusters, those in the atypical symptom cluster—which included anemia and a large percentage of Killip class patients—had worse clinical indicators at hospital readmission during the duration of the 1-year follow-up. Both anemia and high Killip classification suggest that the patient's clinical presentation is poor and therefore the prognosis is worse. Intensive treatment should be considered for anemia and high level of Killip class patients with atypical presentation. Clinicians should focus on patients with atypical symptom clusters, enhance early recognition of symptoms, and develop targeted symptom management strategies to alleviate their discomfort in order to improve symptomatic outcomes.

## 1 Introduction

The American Heart Association (AHA) predicts that about 13 million people worldwide will suffer from cardiovascular disease in 2035, with coronary heart disease (CHD) being the leading cause of death among people with cardiovascular disease (1, 2). The task of preventing and controlling coronary heart disease in China is equally burdensome, and the Report on Cardiovascular Health and Disease in China 2022 points out that the incidence rate of coronary heart disease is rising year by year, and the number of people suffering from coronary heart disease in China has already reached 1.13 million, and the mortality rate of coronary heart disease has reached 0.12%. Since 2005, the mortality rate of coronary heart disease has been rising rapidly and tends to be younger. It can be seen that strengthening the prevention and treatment of coronary heart disease is a key measure to improve the management system of cardiovascular diseases in China (1, 3, 4).

Patients with acute myocardial infarction (AMI) usually present with a variety of symptoms at the onset of the disease, mainly chest pain/chest discomfort, sweating, dyspnea, nausea/vomiting, etc., and present in the form of symptom clusters (5–8). Each patient experiences an average of 3.6–4.75 symptoms in the previous studies (9, 10). Symptom clusters can be defined as two or more concurrently occurring symptoms that interact with each other (11–14). The symptoms of AMI are varied, and multiple symptoms often coexist, creating a negative symptom cluster synergy between symptoms. Compared with single symptoms, symptom clusters can increase the burden of disease in AMI patients, decrease their adherence to treatment, and have a serious negative impact on their quality of life and health status (15–17). The results of related studies have shown that symptom clusters in AMI patients can have a serious impact on functional status, readmission rates for cardiovascular events, and all-cause mortality (18). Symptom clusters consisting of multiple symptoms have a more profound impact on the clinical outcome of patients with AMI than a single symptom (19).

At this stage, the management of symptom clusters in patients with AMI has only progressed to the stage of symptom cluster identification, and more research is needed on the development of symptom cluster management strategies and the evaluation of outcomes (14).

AMI is an aggressive and rapidly progressive condition, and percutaneous coronary intervention (PCI) is the key to its successful treatment and the most common approach for coronary revascularization. Total number of coronary heart disease interventions in China reached 1.16 million in 2021, with 1.48 stents placed per capita in coronary heart disease patients. Reperfusion therapy has a time window requirement, with a 10% increase in patient morbidity and mortality for every 1 h of delay, making early recognition of AMI symptoms and prompt medical attention critical. Problems with the prognosis of PCI also arise; in-stent restenosis, cardiac rupture, and chest pain may cause repeated, multiple admissions to the hospital, which brings a serious economic and psychological burden to patients. It showed nearly 20% of patients were readmitted for cardiovascular events within 1 year of PCI in the previous study (20). Thus unplanned readmission in the early post-procedure period after PCI is considered to be a costly and common adverse outcome (20, 21).

In the United States, readmissions occur in one-fifth of PCI patients each year and account for 33% of the total cost of readmissions, amounting to $26 billion, with a low quality of life and a high economic burden, with 75% of these readmissions considered avoidable. However, there are few studies on the prognostic impact of symptom clusters in AMI patients, and few studies on the impact of symptom clusters on unplanned readmission in AMI patients have been reported. Therefore, the aim of this study was to explore the potential categories of symptom clusters in AMI patients and the effect of potential categories on readmission within 1 year after PCI using latent class analysis, with the aim of helping medical staff to be more targeted in symptom management and providing evidence support and theoretical basis.

## 2 Materials and methods

### 2.1 Study setting and population

This study is a cross-sectional survey study. Convenience sampling method was used to extract patients readmitted for treatment of acute myocardial infarction in the Department of Cardiology of Xianyang City Central Hospital from October 2021 to October 2022 for the study. Inclusion criteria were: (1) patients who survived and those with a final diagnosis of ST-elevation myocardial infraction (STEMI) or Non-ST-elevation myocardial infraction (NSTEMI) who underwent PCI, (2) age 18 years or older, (3) understanding of spoken Chinese, and (4) those who consented to take part in the study. Exclusion criteria were: (1) combination of major organic lesions; (2) new postoperative complications such as myocardial infarction and cerebral infarction.

In this study, the sample size calculation formula was *n* = *Z*^2^_α/2_(1−*P*)*P*/δ^2^, setting the test level α = 0.05(*Z*_α/2 _ = 1.96), with a permissible margin of error δ = 0.05, and based on the results of a similar study in the literature (20), the maximum incidence of re-admission of patients with AMI was 17%, which means that *P* = 0.17, and calculating the sample size *n* = 1.96^2 ^× (1−0.17) × 0.17÷0.05^2 ^= 216 cases, considering the 20% non-response rate, 259 cases were needed for the sample size. A total of 270 patients met the criteria, 9 were dropped due to the long follow-up period and we were unable to contact them. A total of 261 patients were included in this study, with a average age of 62.8 ± 11.2 years (range 34–89), and the rest of the general information, is shown in Table 1.

**Table 1:** Baseline clinical characteristic.

Variables	Classification	*N* = 261	%
Age (years)	34–60	109	41.8
	61–89	152	58.2
Final diagnosis	ST-elevation myocardial infraction	178	68.2
	Non-ST-elevation myocardial infraction	83	31.8
Gender	Female	42	16.1
Marital status	Divorced/widowed/never married	2	0.8
Occupation status	Unemployed/retired	38	14.6
Risk factors^a^	Hypertension	120	46.0
	Diabetes mellitus	47	18.0
	Dyslipidemia	48	18.4
	Current smoking	135	51.7
	Obesity = body mass index>28 kg/m^2^	24	9.2

^a^ Answers were duplicated.

### 2.2 Ethics statement

The study protocol was approved by the Ethical Research Board of the affiliated institution (IRB No. 2023-64). Patients who consented to take part in the study signed an informed consent. Patient readmission information was obtained by telephone interview during follow-up period.

### 2.3 Questionnaire and data collection

#### 2.3.1 Patients characteristics

The questionnaire of patients characteristics was developed by the researcher and consisted of 3 parts, (1) sociodemographic characteristic: gender, age, working status, marital status, and mode of payment for medical care; (2) disease-related information: risk factors, diagnosis, comorbid conditions, cardiac function class, Killip class, type of coronary artery lesion, history of hemodialysis and total hospital stay and (3) clinical outcome information: readmission within 1 year.

#### 2.3.2 AMI symptoms

For this study, Illness Perception Questiormaire Revised (IPQ-R) was selected to collect patients symptoms. The IPQ-R is a targeted assessment tool for assessing the multisymptomatic nature of AMI and was developed by Moss Morris in a revision of the IPQ developed by Weinman et al. There are 11 symptoms, including: (1) chest pain, (2) chest discomfortable, (3) radiating pain, (4) dyspnea, (5) palpitation, (6) nausea/vomiting, (7) sweating, (8) weakness/fatigue, (9) dizziness, (10) syncope, (11) throat tighting sensation. Cronbach's α coefficient for internal consistency was 0.66–0.92 in the previous studies (22–25).

#### 2.3.3 Hospital readmission

Unplanned readmission is defined as any unpredictable readmission that occurs after discharge for an activity that is not performed within the normal plan, excluding planned readmissions (e.g., follow-up review, secondary surgery, etc.) (26). Retain contact information and verify contact information for patients and their families during their hospitalization. We obtained readmission information through telephone interviews with patients by asking about the date and reason for readmission in the past 1 year. Hospital readmission was defined as a return or visit to the hospital due to AMI related complications or AMI recurrence, deterioration within 12 months after hospital discharge for AMI. Readmission excluded planned readmissions, such as elective surgery, regular hospital admissions for treatment, review, etc. and unrelated to the reason for the last hospitalization.

The purpose and requirements of the study were explained to the patients who met the criteria by two investigators who were uniformly trained, and the questionnaire was administered within 3 days of the patient's admission after obtaining informed consent. Uniform training was given to the investigators to ensure that they conducted one-on-one on-site surveys using uniform instructions; when data were collected face-to-face, they were filled in by the research subjects themselves; for older patients, they could be filled in on behalf of the research subjects by asking them one by one; if there were any questions during the filling in process, the researchers provided timely explanations, the questionnaires were distributed and collected on the spot, and on-site verification was carried out instantly. If there is any omission or obvious error, the research subjects are reminded to make up or correct in time; the questionnaire number is entered in time, and the confidentiality of the information is done. Each patients was collected information about the symptoms, risk factors, and chronic diseases related to the acute phase of acute myocardial infarction. Disease-related information was collected by reviewing the medical record system. Data for clinical outcome including hospital readmission was also obtained.

### 2.4 Statistical analysis

Latent class analysis (LCA) was used for analyzing the coded symptoms, and the evaluation indexes were as follows. (1) Information criteria: Akaike Information Criterion (AIC), Bayesian Information Criterion (BIC) and adjusted Bayesian Information Criterion (aBIC). Smaller values of such information criteria indicate better model fit. (2) Entropy index: indicates the degree of classification accuracy, the value range is 0–1, the closer to 1 indicates that the classification is more accurate. (3) Likelihood ratio metrics: Lo-Mendell-Rubin (LMR) and Bootstrapped likelihood ratio test (BLRT), significant LMR, BLRT values indicate that a model with K categories outperforms a model with K-1 categories (27, 28).

The collected data were analyzed using SPSS Statistic v.24. Univariate analysis was used to compare differences in the characteristics of samples of patients with different symptom clusters. Multiple logistic regression analysis was used to identify the effect of symptom clusters on readmission after adjusting for patients’ baseline characteristics. The statistic significance level(α) was set at 0.05.

## 3 Results

### 3.1 Three sets of symptoms were identified by latent cluster analysis

Patients were included in the potential category model, and a total of 5 models were fitted. In model 3, AIC and aBIC were the smallest, the *P*-value of BLRT test was <0.05, and the entropy was >0.7. Based on the clinical experience, model 3 was chosen to categorize the patients into 3 categories, as shown in Table 2.

**Table 2:** Model fit results of LCA for symptom cluster in acute myocardial patients (*n* = 261).

Models	AIC	BIC	aBIC	Entropy	*P*		Number of patients in each category
					LMR-LRT	BLRT	
1	2,217.636	2,256.846	2,221.971	—	—	—	—
2	2,080.710	2,162.694	2,089.774	1.000	<0.001	<0.001	79/182
3	2,063.825	2,188.583	2,077.618	0.882	0.141	<0.001	168/77/16
4	2,066.800	2,234.332	2,085.323	0.800	0.387	0.158	48/5/80/128
5	2,069.524	2,279.831	2,092.776	0.839	0.148	0.500	158/19/13/10/61

### 3.2 Significant differences in the number and composition of the symptom in 3 cluster groups

Cluster 1 had the highest number of participants (*n* = 168, 64.4%), followed by Cluster 2 (*n* = 77, 29.5%) and Cluster 3 (*n* = 16, 6.1%).On average, patients reported 2.9 ± 1.2 symptoms. There was a significant difference in the number of symptoms among the 3 clusters (*P* < 0.001); with the highest number of symptoms in cluster 3 (5.0 ± 2.9) and the lowest number of symptoms in cluster 1 (2.6 ± 1.2). As for the distribution of symptoms in each cluster, excluding nausae or vomiting, sweating and syncope, the remaining symptoms (chest pain, chest discomfortable, pain or discomfortable in other parts, shortness of breath, palpitation, weakness or fatigue [all *P* < 0.001]; dizziness [*P* = 0.014]; throat tightening sensation [*P* = 0.036) showed significant differences between clusters. Most common was chest pain (83.9%), followed by sweating (73.2%). Less frequently reported symptoms were syncope (7.3%), palpitation (2.7%), and throat tightening sensation (1.9%) (Table 3).

**Table 3:** Comparison of frequency of presenting symptoms by cluster group **(***n* = 261).

	Total	Cluster 1 (*n* = 168)	Cluster 2 (*n* = 77)	Cluster 3 (*n* = 16)	*F*/*χ^2^*	*P*
No of total symptom (mean ± SD)	2.9 ± 1.2	2.6 ± 1.2	3.1 ± 1.1	5.0 ± 2.9	13.559	<0.001
Chest pain	219 (83.9)	168 (100.0)	44 (57.1)	7 (43.8)	99.475	<0.001
Chest discomfortable	79 (30.3)	0	76 (98.7)	3 (18.8)	285.615	<0.001
Radiating pain	57 (21.8)	37 (22.0)	8 (10.4)	12 (75.0)	27.035	<0.001
Dyspnea	92 (35.2)	25 (14.9)	60 (77.9)	7 (43.8)	92.476	<0.001
Palpitation	7 (2.7)	1 (6.0)	0	6 (37.5)	29.406	<0.001
Nausea/vomiting	67 (25.7)	43 (25.6)	16 (20.8)	8 (50.0)	5.499	0.059
Sweating	191 (73.2)	126 (75.0)	53 (68.8)	12 (75.0)	1.086	0.616
Weakness/fatigue	13 (5.0)	1 (6.0)	3 (3.9)	9 (56.3)	43.377	<0.001
Dizziness	19 (7.3)	8 (4.8)	7 (9.1)	4 (25.9)	7.819	0.014
Syncope	9 (3.4)	6 (3.6)	3 (3.9)	0	0.183	1.000
Throat tighting sensation	5 (1.9)	2 (1.2)	1 (1.3)	2 (12.5)	6.230	0.036

**Figure 1:**
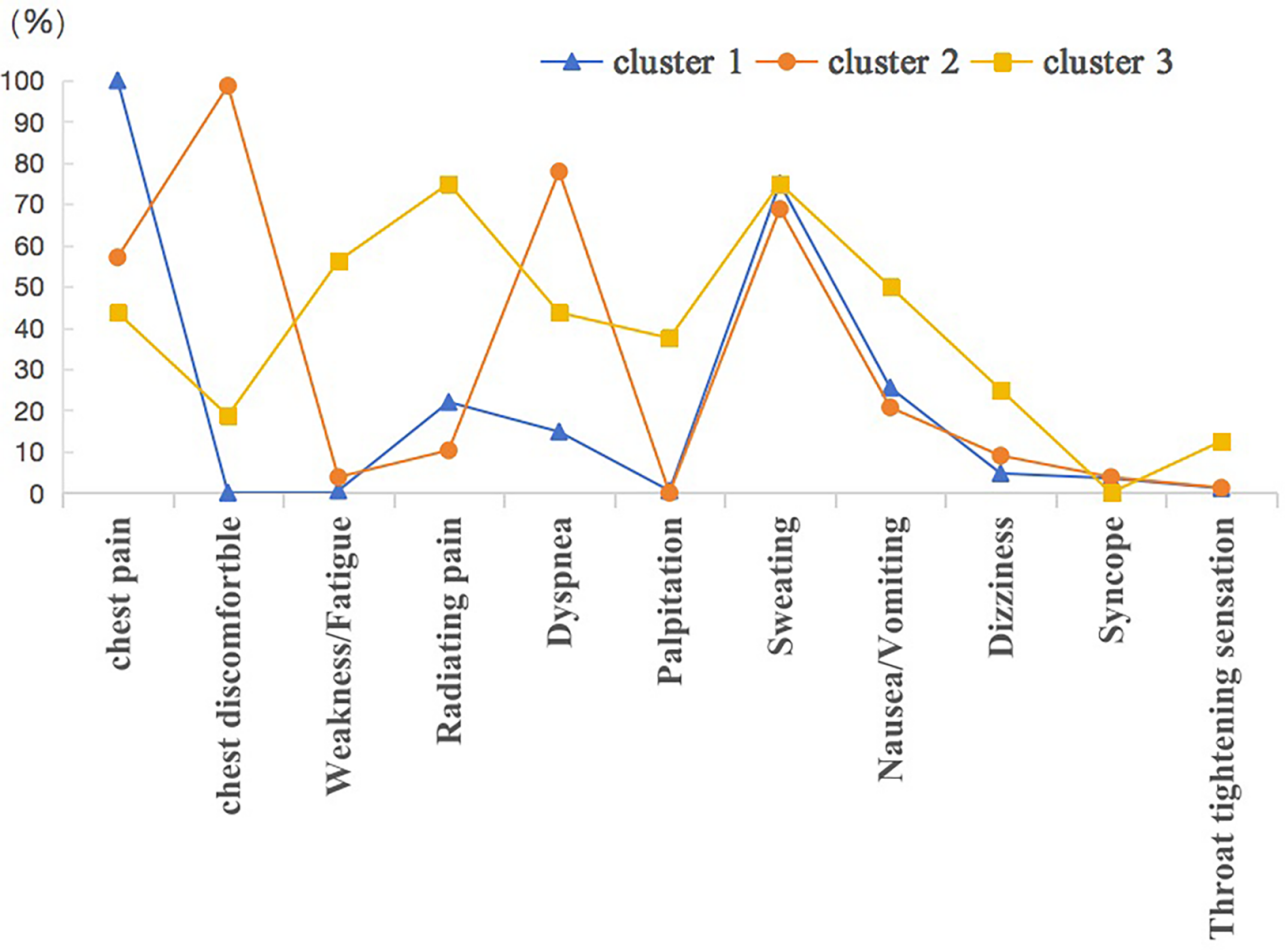
Symptom distribution by clustered groups.

In cluster 1, the frequency of chest pain was the highest at 100%, followed by sweating (75.0%) and nausea or vomiting (25.6%). In cluster 2, chest discomfortable at 98.7%, followed by shortness of breath (77.9%) and sweating (68.8%). Many atypical symptoms were present in Cluster 3, including radiating pain (75.0%), weakness or fatigue (56.3%), palpitation (37.5%) and dizziness (25.9%). The clusters have been called “classic symptoms” (cluster 1), “multiple symptoms” (cluster 2), and “atypical symptoms” (cluster 3), taking into account the number of symptoms, frequency, and distribution (Figure 1).

### 3.3 Patients of cluster 3 are more likely to have anemia and be with high value of killip class

In terms of comorbidities, the results of the univariate analysis in this study showed that there were differences in anemia (*P* = 0.016) among patients with different potential categories of AMI symptom clusters, and the patients of atypical symptom cluster were more likely to have anemia.

For disease factors, the results of the univariate analysis in this study showed that there were differences in Killip classes (*P* = 0.003) in patients with different potential categories of AMI symptom clusters, and the patients of atypical symptom clusters were more likely to have higher value for Killip classes, which has severe heart failure (Table 4).

**Table 4:** Relationships between cluster membership and sample characteristics (*n* = 261).

Variables	Cluster 1	Cluster 2	Cluster 3	*P*
	168 (64.4%)	77 (29.5)	16 (6.1)	
Age (years)				
(mean ± SD)	62.5 ± 11.1	63.3 ± 11.1	63.3 ± 12.5	0.879
<60	68	27	6	0.718
≥60	100	50	10	
Gender, *n* (%)				
Male	145 (86.3)	59 (76.6)	15 (93.8)	0.105
Female	23 (13.7)	18 (23.4)	1 (6.2)	
Occupation, *n* (%)				
Employed	146 (86.9)	65 (84.4)	12 (75.0)	0.368
Unemployed/retired	22 (13.1)	12 (15.6)	4 (25.0)	
Marital status, *n* (%)				
Married	167 (99.4)	76 (98.7)	16 (100.0)	0.587
Divorced/widowed/never married, *n* (%)	1 (0.6)	1 (1.3)	0	
Insurance type, *n* (%)				
Insurance	165 (98.2)	77 (100.0)	15 (93.8)	0.205
Self-paying	3 (1.8)	0	1 (6.2)	
Smoking, *n* (%)				
None	63 (37.5)	36 (46.8)	4 (25.0)	0.376
Current	91 (54.2)	34 (44.2)	10 (62.5)	
Former	14 (8.3)	7 (9.1)	2 (12.5)	
Hypertension, *n* (%)	82 (48.8)	34 (44.2)	4 (25.0)	0.176
Dyslipidemia, *n* (%)	29 (17.3)	17 (22.1)	2 (12.5)	0.594
Diabetes mellitus, *n* (%)	27 (16.1)	16 (20.8)	4 (25.0)	0.435
Obesity (body mass index>28 kg/m^2^), *n* (%)	15 (8.9)	9 (11.7)	0	0.419
Diagnosis STEMI, *n* (%)	114 (67.9)	51 (66.2)	13 (81.3)	0.496
NSTEMI, *n* (%)	54 (32.1)	26 (33.8)	3 (18.7)	
New onset heart failure in hospital, *n* (%)	8 (4.8)	3 (3.9)	0	1.000
History of revascularization, *n* (%)	5 (3.0)	2 (2.6)	2 (12.5)	0.171
Combidity, *n* (%)				
Coronary heart disease	9 (5.4)	3 (3.9)	3 (18.8)	0.097
Atrial fibrillation	10 (6.0)	1 (1.3)	2 (12.5)	0.084
Chronic heart failure	0	1 (1.3)	0	0.356
Cerebrovascular disease	15 (8.9)	8 (10.4)	1 (6.3)	0.934
Peripheral arterial disease	1 (0.6)	0	0	1.000
COPD	1 (0.6)	2 (2.6)	1 (6.3)	0.075
Chronic kidney disease	1 (0.6)	0	1 (6.3)	0.119
Anemia	7 (4.2)	4 (5.2)	4 (25.0)	0.016
Thyroid dysfunction	6 (3.6)	1 (1.3)	2 (12.5)	0.123
Malignancy	0	0	1 (6.3)	0.061
Numbers of symptoms, *n* (%)	2.6 ± 1.2	3.1 ± 1.1	5.0 ± 2.9	<0.001
Killip class≥Ⅱ, *n* (%)	42 (25.0)	30 (39.0)	9 (56.3)	0.003
Types of coronary artery disease, *n* (%)				
Single-vessel disease	26 (15.5)	7 (9.1)	2 (12.5)	0.434
Multi-vessel disease	142 (84.5)	70 (90.9)	14 (87.5)	
Total hospital stay (days)	7.0 ± 2.3	7.6 ± 2.7	7.9 ± 2.7	0.07

STEMI:ST-elevation myocardial infraction,NSTEMI:Non-ST-elevation myocardial infraction.

### 3.4 Patients in cluster 3 anticipated a noticeably higher risk of 1-year readmission

During the follow-up period, a total of 55 (21.1%) patients had readmissions, including 23 (8.8%) planned readmissions, 4 (1.5%) unrelated disease readmissions, and 28 (10.7%) unplanned readmissions.

A total of 28 patients readmitted during the course of the follow-up period. The hospital readmission proved to be significantly varied by cluster, using bivariate analysis (*P* = 0.001) (Table 5). Multinomial logistic regression analysis demonstrated that when patients characteristic were adjusted for, compared to patients in Cluster 1, patients in Cluster 3 anticipated a noticeably higher risk of 1-year readmission (odd ratio 8.303, 95% confidence interval 2.550–27.031, *P* < 0.001) (Table 6).

**Table 5:** Hospital readmission at 1-year follow-up by symptom clusters.

	Cluster 1 (*n* = 168)	Cluster 2 (*n* = 77)	Cluster 3 (*n* = 16)	*P*
Readmission				
0	155 (92.3)	69 (89.6)	9 (56.3)	0.001
≥1	13 (7.7)	8 (10.4)	7 (43.7)	

**Table 6:** Multinomial logistic regression analysis:factors related to each cluster.

Variables	B	SE	OR	*P*	95% CI	
					Lower	Upper
Cluster 2 vs. Cluster 1	0.206	0.490	1.228	0.675	0.470	3.209
Cluster 3 vs. Cluster 1	2.117	0.602	8.303	<0.001	2.550	27.031

## 4 Discussion

This study identified symptom clusters in patients with AMI, explored the relationship between symptom clusters and patient-related variables, and examined into the impact of symptom clusters on hospital readmission in order to develop intervention strategies to lower the risk of readmission in Chinese patients with AMI. Using the latent class analysis, three symptom clusters were identified: typical chest symptom cluster (chest pain, sweating, nausea/vomiting), multiple symptom cluster(chest discomfortable, shortness of breath, sweating) and atypical symptom cluster(radiating pain, weakness/fatigue, palpitation, dizziness). Previous studies have categorized symptoms into four to five clusters (9, 19), with differences in this classification based on factors such as the target population, inclusion criteria, number of symptoms evaluated, and methods of clustering (29).

Hwang et al. (18) used latent class cluster analysis to extract three clusters were: typical chest pain (chest pain/discomfortable, cold sweat, shortness of breath, nause/vomiting, weakness/fatigue/dizziness), multiple symptoms (chest pain/discomfortable, left shoulder/arm pain, cold sweat, right shoulder/arm pain, nausea/vomiting), atypical symptom (shortness of breath, weakness/fatigue/dizziness, indigestion/abdominal pain, cold sweat, nausea/vomiting).

The results of the two studies were basically the same, but the composition of the symptoms within the clusters was not exactly the same, and the reasons for this were analyzed which were related to the inconsistency of the symptoms collected. This study used the IPQ-R with 11 symptom entries, Huwang et al. (18) analyzed 14 symptom entries and the composition of the entries was inconsistent.

Kim et al. (16) used cluster analysis to extract three symptom clusters, namely classic MI, stress symptoms, and multiple symptoms. In comparison to the present study, both of them yielded the same number of symptom clusters, although they took different statistical methods.

McSweeney et al. (30) used cluster analysis on 1,270 female patients, which includes 37 acute MI symptoms, to extract a total of 3 symptom clusters, (1) old, silent asymptomatic group, (2)diverse, mildly symptomatic group, and (3) younger, minority, multiple distressing symptom group.

In comparison to the present study, the number of symptom clusters derived from the two were the same despite different statistical methods, but the number of symptoms comprising the clusters was greater in the study by McSweeney et al. (30) than in the present study. The reason for this is that the questionnaire used by McSweeney et al. had 37 symptom entries, which is more than the 11 entries in the present study.

In cluster 1 (typical chest symptom cluster), chest pain and sweating were predominant. This is similar to the “older/silent asymptomatic” reported by McSweeney et al. (30) and to the “classic symptoms” in the study by Lindgren et al. (31). The cluster 1 is characterized by a high incidence of typical chest symptoms in the previous studies (16, 30, 32, 33).

In cluster 2 (multiple symptom cluster), chest discomfortable and dyspnea were predominant. Cluster 2 in our study is similar to the “heavy symptom burden”, and “chest pain”, “shortness of breath” and “sweating” were the main components in the study by Devon et al. (34). Cluster 2 was characterized by a high prevalence of each symptom, including chest symptoms and other symptoms.

In cluster 3 (atypical symptom cluster), radiating pain, sweating and weakness/fatigue were predominant. The characteristics of cluster 3 are in part similar to the “weary symptom” reported by Rosenfeld et al. (33). Cluster 3 was characterized by a wide variety of symptoms but no representative symptoms with a high incidence, and a low incidence or absence of chest symptoms. Cluster 3 can lead to delays in seeking medical care due to the lack of typical chest pain symptoms and the inability of patients to recognize AMI symptoms in a timely manner or failing to attribute them to cardiac disease (35–37).

In this study, we found that in terms of the prevalence of symptoms, the presence of 8 of the 11 symptoms on the symptom assessment form was greater than 20% in patients with AMI at the time of admission, with the prevalence of chest pain, sweating, dyspnea, discomfort in the chest, nausea/vomiting, and radiating pain in descending order of prevalence, among which the prevalence of chest pain and cold sweating was greater than 50%.

This is similar to the findings of Kim et al. Kim found that the top 6 symptoms in terms of symptom prevalence in a survey of STEMI patients at the time of hospital admission were chest pain, sweating, dyspnea, nausea or vomiting, weakness radiating pain and dizziness. The results of Kim's study are almost included in terms of symptom composition, but the difference is that the symptoms of weakness and dizziness were ranked more highly in Kim's study. The reason for this analysis is related to the fact that the AMI patients investigated in this study included NSTEMI patients, whereas the Kim investigations were all STEMI patients, and when compared to the two, the STEMI patients had large myocardial infarctions and were more prone to arrhythmias, and therefore had weakness and dizziness as their main manifestations.

Because of the existence of insufficient tissue perfusion in STEMI patients, the body is in a state of ischemia and hypoxia, so they show a state of weakness/dizziness, while NSTEMI patients have a lower incidence of weakness/dizziness than STEMI patients due to the longer duration of the lesion and the prolonged presence of ischemia and hypoxia, which is somewhat tolerated by the body.

In terms of comorbidities, the results in this study showed that there were differences in anemia among patients with different latent classes of AMI symptom clusters, and that patients with anemia are more likely to fall into the atypical symptom cluster. Anemia is a common comorbidity of AMI (38). Anemia exacerbates coronary artery ischemia, reduces oxygen-carrying capacity and myocardial oxygen consumption, and patients with anemia have a low hemoglobin concentration and a decreased ability of red blood cells to transport oxygen, which further aggravates the originally damaged myocardial ischemia and hypoxia, deforms cardiomyocytes, and receives a severe impact on cardiac systolic and diastolic function. The symptoms of weakness or fatigue and dizziness in the atypical symptom cluster may be related to anemia.

The atypical symptom cluster had considerably higher Killip class levels. Patients with higher Killip classification have more severe heart failure and poor clinical performance, and it has been shown that high Killip classification is an independent predictor of mortality in STEMI and NSTEMI in previous study (39). One explanation for this seems to be that patients with atypical symptom clusters have atypical symptoms, which are more likely to be delayed or misdiagnosed, resulting in more severe heart failure and thus affecting the prognosis. This study shows that atypical symptom clusters are predictors of whether readmission occurs in AMI patients.

Atypical symptom cluster related with significantly higher risk of readmission than typical chest symptom cluster and multiple symptom cluster. The findings of Hwang et al. (40) found that patients with the presence of atypical symptom clusters had the highest incidence of major adverse cardiovascular events (MACE) within 12 months. Hwang et al. (18) showed that the risk of death within 1 year in patients with the presence of atypical symptom clusters was 3.3 times. The results of related studies suggest that atypical symptom clusters in AMI patients can seriously affect the all-cause mortality of patients (11). The above findings suggest that atypical symptom cluster is an significant predictor of poor clinical outcomes in AMI patients, which may be related to the lack of typical chest pain symptoms, affecting patients’ recognition of the disease and leading to delayed access to medical care, which in turn leads to a poor prognosis (11, 31, 41). Therefore, patients with atypical symptoms on admission should not only be treated properly during hospitalization, but also should not be neglected in post-discharge follow-up.

Patients with atypical symptom clusters are more likely to experience delayed consultation or be misdiagnosed than those with typical symptom clusters. Early recognition, diagnosis, and treatment of the disease facilitates accelerated recovery and shortens the duration of painful symptom, thereby improving symptom experience. Thus, providing patients with awareness and coping skills of AMI symptoms and symptom clusters is conducive to symptom relief. David et al. (42) combined health education and skill development, and through the implementation of health education and skill development interventions for females hospitalized for ACS, instructed the patients in symptom monitoring and analysis, recognition of symptom patterns and adoption of appropriate coping. The effectiveness of this intervention was confirmed. This shows that health education and skill development interventions can improve the ability to recognize and respond to the symptoms and symptom clusters of female patients with ACS, so that the patients can receive effective diagnosis and treatment.

Traditional Chinese medicine (TCM) has been applied as a symptom-relieving intervention for symptom management in patients with ACS and has been shown to be effective. It has been shown that auricular point pressure beans in combination with acupoint has a relieving effect on symptom clusters related to chest pain in patients with ACS (43). In the future, we can link up with the Traditional Chinese medicine to explore its long-term effect on symptom clusters.

Coronary artery disease is relatively costly to treat, may require long-term medication, and carries a high healthcare burden for patients. Therefore, the application value of the results of this study in clinical care is to screen high-risk readmission patients, and in the future, pre-discharge and post-discharge interventions can be developed for high-risk readmission patients, including medication use, self-management, follow-up, and monitoring of patients’ disease progression, in order to enhance the quality of life of the patients, to further reduce the rate of in-admission, to reduce the burden of medical care for readmission, and to provide a follow-up continuity of care and a rehabilitation program ideas.

### 4.1 Limitations and suggestion

First, although the sample size is more than sufficient to meet the needs of the study, only one hospital was selected, and the hospital under investigation was a national cardiovascular hospital. The diagnosis and service level of the hospital is high, and the cooperation of the investigated patients is high, which has certain special characteristics, and the sample still lacks sufficient representativeness despite the fact that the investigated hospital's consultation area radiates the northwest region of China. There is a problem of readmission rate bias, patients may have other choices of medical treatment, which may underestimate the readmission rate. It is suggested that future studies should expand the sample size, select AMI patients from different levels of hospitals in different regions, and consider conducting relevant international cooperative studies to compare the differences in symptom clusters of AMI patients from different countries and ethnic groups, in order to discover the pattern of change of symptom clusters, and to help clinical nurses guide patients to manage their symptoms in a more scientific way.

Secondly, the follow-up period was only 1 year after the patients were discharged from the hospital, and future studies may explore the symptom clusters at different time points after discharge to clarify the trajectory changes of the symptom clusters, which may suggest that we adopt different diagnostic and therapeutic measures according to the different phases, in order to improve the prognosis of the patients and enhance the quality of survival.

Another limitation is that follow-up visits are conducted by telephone, which has limitations in obtaining information about the patient, mainly because of the subjectivity of the patient's answers. In the future, consideration will be given to incorporating cell phone software in the form of self-assessment of the patient under permissible conditions, or using remote monitoring equipment to objectively and dynamically understand the patient's condition. In the future, remote network technology can also be combined with symptom management strategies to dynamically monitor patients’ symptoms in real time and analyze subgroups of symptom clusters to help clinical caregivers identify high-risk patients and provide targeted guidance.

Finally, latent class analysis is only a method of extracting symptom clusters, and future research can use cluster analysis, exploratory factor, symptom cluster subgroup analysis, and compare the above methods, which is more in line with the patient's actual situation, with a view to helping clinical caregivers to accurately grasp the patient's symptom characteristics, so as to implement targeted care measures.

## 5 Conclusions

We used latent class analysis to identify three potential categories of AMI patients, which were typical chest symptom clusters, atypical symptom clusters, and multiple symptom clusters. Clinical data were collected on a total of 261 patients, of whom 28 patients experienced unplanned readmission within 1 year, a readmission rate of 10.7%. Anemia and Killip's classification were the most important factors influencing the potential categories of symptom clusters of the patients, and the patients with atypical symptom clusters were at higher risk of readmission within 1 year than the patients with other symptom clusters. Healthcare providers can pay more attention to patients with atypical symptom clusters, combining patients’ symptoms with clusters for efficient and precise symptom management to improve patients’ health outcomes. We recommended that future longitudinal studies explore the symptom clusters at different time points and clarify the trajectory of the symptom clusters, in order to guide the healthcare providers to adopt different diagnostic and therapeutic measures according to the different stages to improve the prognosis of the patients.

## Data Availability

The original contributions presented in the study are included in the article/Supplementary Material, further inquiries can be directed to the corresponding authors.

## References

[1] Tsao CW Aday AW Almarzooq ZI Anderson CAM Arora P Avery CL Heart disease and stroke statistics-2023 update: a report from the American Heart Association. Circulation. (2023) 147(8):e93–e621. 10.1161/CIR.0000000000001123 36695182 PMC12135016

[2] Ding X Zhang Y Li J Mao B Guo Y Li G. A feasibility study of multi-mode intelligent fusion medical data transmission technology of industrial internet of things combined with medical internet of things. IoT. (2023) 21:100689. 10.1016/j.iot.2023.100689

[3] Ades PA. Cardiac rehabilitation and secondary prevention of coronary heart disease. N Engl J Med. (2001) 345(12):892–902. 10.1056/NEJMra001529 11565523

[4] Zhang X Mao B Che Y Kang J Luo M Qiao A Physics-informed neural networks (PINNs) for 4D hemodynamics prediction: an investigation of optimal framework based on vascular morphology. Comput Biol Med. (2023) 164:107287. 10.1016/j.compbiomed.2023.107287 37536096

[5] Dodd MJ Miaskowski C Paul SM. Symptom clusters and their effect on the functional status of patients with cancer. Oncol Nurs Forum. (2001) 28(3):465–70. 11338755

[6] Kim HJ McGuire DB Tulman L Barsevick AM. Symptom clusters: concept analysis and clinical implications for cancer nursing. Cancer Nurs. (2005) 28(4):270–82. 10.1097/00002820-200507000-00005 16046888

[7] Aktas A. Cancer symptom clusters: current concepts and controversies. Curr Opin Support Palliat Care. (2013) 7(1):38–44. 10.1097/SPC.0b013e32835def5b 23287418

[8] Li G Wang H Zhang M Tupin S Qiao A Liu Y Prediction of 3D cardiovascular hemodynamics before and after coronary artery bypass surgery via deep learning. Commun Biol. (2021) 4(1):99. 10.1038/s42003-020-01638-1 33483602 PMC7822810

[9] Gao Y Zhang HJ. The effect of symptoms on prehospital delay time in patients with acute myocardial infarction. J Int Med Res. (2013) 41(5):1724–31. 10.1177/0300060513488511 23926196

[10] Horne R James D Petric K Weinman J Vincent R. Patients’ interpretation of symptoms as a cause of delay in reaching hospital during acute myocardial infarction. Heart. (2000) 83(4):388–93. 10.1136/heart.83.4.388 10722534 PMC1729385

[11] Riegel B Hanlon AL McKinley S Moser DK Meischke H Doering LV Differences in mortality in acute coronary syndrome symptom clusters. Am Heart J. (2010) 159(3):392–8. 10.1016/j.ahj.2010.01.003 20211300 PMC2844635

[12] Barsevick AM Whitmer K Nail LM Beck SL Dudley WN. Symptom cluster research: conceptual, design, measurement, and analysis issues. J Pain Symptom Manage. (2006) 31(1):85–95. 10.1016/j.jpainsymman.2005.05.015 16442485

[13] Dodd M Janson S Facione N Faucett J Froelicher ES Humphreys J Advancing the science of symptom management. J Adv Nurs. (2001) 33(5):668–76. 10.1046/j.1365-2648.2001.01697.x 11298204

[14] Miaskowski C Dodd M Lee K. Symptom clusters: the new frontier in symptom management research. J Natl Cancer Inst Monogr. (2004) 32:17–21. 10.1093/jncimonographs/lgh023 15263036

[15] Birnbach B Höpner J Mikolajczyk R. Cardiac symptom attribution and knowledge of the symptoms of acute myocardial infarction: a systematic review. BMC Cardiovasc Disord. (2020) 20(1):445. 10.1186/s12872-020-01714-8 33054718 PMC7557019

[16] Kim HS Eun SJ Hwang JY Lee KS Cho SI. Symptom clusters and treatment time delay in Korean patients with ST-elevation myocardial infarction on admission. Medicine (Baltimore). (2018) 97(19):e0689. 10.1097/MD.0000000000010689 29742716 PMC5959405

[17] Li G Song X Wang H Liu S Ji J Guo Y Prediction of cerebral aneurysm hemodynamics with porous-medium models of flow-diverting stents via deep learning. Front Physiol. (2021) 12:733444. 10.3389/fphys.2021.733444 34603085 PMC8484706

[18] Hwang SY Ahn YG Jeong MH. Atypical symptom cluster predicts a higher mortality in patients with first-time acute myocardial infarction. Korean Circ J. (2012) 42(1):16–22. 10.4070/kcj.2012.42.1.16 22363379 PMC3283750

[19] Yu DS Li PW Chong SO. Symptom cluster among patients with advanced heart failure: a review of its manifestations and impacts on health outcomes. Curr Opin Support Palliat Care. (2018) 12(1):16–24. 10.1097/SPC.0000000000000316 29176333

[20] Oliveira L Costa I Silva D Silva J Barreto-Filho J Almeida-Santos M Readmission of patients with acute coronary syndrome and determinants. Arq Bras Cardiol. (2019) 113(1):42–9. 10.5935/abc.20190104 31271598 PMC6684196

[21] Kwok CS Chatterjee S Bagur R Sharma K Alraies MC Fischman D Multiple unplanned readmissions after discharge for an admission with percutaneous coronary intervention. Catheter Cardiovasc Interv. (2021) 97(3):395–408. 10.1002/ccd.28797 32108416

[22] Sir Ö Özakgül A. Evaluation of the perception of illness and quality of life in patients with acute myocardial infarction. Turk Kardiyol Dern Ars. (2022) 50(3):209–16. 10.5543/tkda.2022.21048 35450845

[23] Yu Y Wu AMS Wing YK Chan JWY Lau MMC Lau JTF. Validation of the revised illness perception questionnaire of obstructive sleep apnea among elderly Chinese in the general population. Sleep Breath. (2023) 27(1):337–44. 10.1007/s11325-022-02598-y 35377089

[24] Villalobos-Galvis FH Mafla AC Burbano-Trujillo WF Sanchez-Figueroa AA. Psychometric properties of the revised illness perception questionnaire for oral health. Caries Res. (2017) 51(3):244–54. 10.1159/000468993 28501863

[25] Rivera E Levoy K Clark-Cutaia MN Schrauben S Townsend RR Rahman M Content validity assessment of the revised illness perception questionnaire in CKD using qualitative methods. Int J Environ Res Public Health. (2022) 19(14):8654. 10.3390/ijerph19148654 35886505 PMC9319998

[26] Wasfy JH Zigler CM Choirat C Wang Y Dominici F Yeh RW. Readmission rates after passage of the hospital readmissions reduction program: a pre-post analysis. Ann Intern Med. (2017) 166(5):324–31. 10.7326/M16-0185 28024302 PMC5507076

[27] Yang Q Zhao A Lee C Wang X Vorderstrasse A Wolever RQ. Latent profile/class analysis identifying differentiated intervention effects. Nurs Res. (2022) 71(5):394–403. 10.1097/NNR.0000000000000597 35417442

[28] MacLean E Dendukuri N. Latent class analysis and the need for clear reporting of methods. Clin Infect Dis. (2021) 73(7):e2285–6. 10.1093/cid/ciaa1131 32761073

[29] DeVon HA Vuckovic K Ryan CJ Barnason S Zerwic JJ Pozehl B Systematic review of symptom clusters in cardiovascular disease. Eur J Cardiovasc Nurs. (2017) 16(1):6–17. 10.1177/1474515116642594 27034451

[30] McSweeney JC Cleves MA Zhao W Lefler LL Yang S. Cluster analysis of women’s prodromal and acute myocardial infarction symptoms by race and other characteristics. J Cardiovasc Nurs. (2010) 25(4):311–22. 10.1097/JCN.0b013e3181cfba15 20539165 PMC2884391

[31] Lindgren TG Fukuoka Y Rankin SH Cooper BA Carroll D Munn YL. Cluster analysis of elderly cardiac patients’ prehospital symptomatology. Nurs Res. (2008) 57(1):14–23. 10.1097/01.NNR.0000280654.50642.1a 18091288

[32] Ryan CJ DeVon HA Horne R King KB Milner K Moser DK Symptom clusters in acute myocardial infarction: a secondary data analysis. Nurs Res. (2007) 56(2):72–81. 10.1097/01.NNR.0000263968.01254.d6 17356437

[33] Rosenfeld AG Knight EP Steffen A Burke L Daya M DeVon HA. Symptom clusters in patients presenting to the emergency department with possible acute coronary syndrome differ by sex, age, and discharge diagnosis. Heart Lung. (2015) 44(5):368–75. 10.1016/j.hrtlng.2015.05.008 26118542 PMC4567428

[34] DeVon HA Ryan CJ Rankin SH Cooper BA. Classifying subgroups of patients with symptoms of acute coronary syndromes: a cluster analysis. Res Nurs Health. (2010) 33(5):386–97. 10.1002/nur.20395 20672306 PMC3102439

[35] Farshidi H Rahimi S Abdi A Salehi S Madani A. Factors associated with Pre-hospital delay in patients with acute myocardial infarction. Iran Red Crescent Med J. (2013) 15(4):312–6. 10.5812/ircmj.2367 24083004 PMC3785905

[36] Thuresson M Jarlöv MB Lindahl B Svensson L Zedigh C Herlitz J. Thoughts, actions, and factors associated with prehospital delay in patients with acute coronary syndrome. Heart Lung. (2007) 36(6):398–409. 10.1016/j.hrtlng.2007.02.001 18005801

[37] Moser DK Kimble LP Alberts MJ Alonzo A Croft JB Dracup K Reducing delay in seeking treatment by patients with acute coronary syndrome and stroke: a scientific statement from the American heart association council on cardiovascular nursing and stroke council. J Cardiovasc Nurs. (2007) 22(4):326–43. 10.1097/01.JCN.0000278963.28619.4a 17589286

[38] Salisbury AC Alexander KP Reid KJ Masoudi FA Rathore SS Wang TY Incidence, correlates, and outcomes of acute, hospital-acquired anemia in patients with acute myocardial infarction. Circ Cardiovasc Qual Outcomes. (2010) 3(4):337–46. 10.1161/CIRCOUTCOMES.110.957050 20488919 PMC3384714

[39] El-Menyar A Zubaid M AlMahmeed W Sulaiman K AlNabti A Singh R Killip classification in patients with acute coronary syndrome: insight from a multicenter registry. Am J Emerg Med. (2012) 30(1):97–103. 10.1016/j.ajem.2010.10.011 21159479

[40] Hwang SY Kim J. Cluster dyads of risk factors and symptoms are associated with major adverse cardiac events in patients with acute myocardial infarction. Int J Nurs Pract. (2015) 21(2):166–74. 10.1111/ijn.12241 24593680

[41] Canto JG Shlipak MG Rogers WJ Malmgren JA Frederick PD Lambrew CT Prevalence, clinical characteristics, and mortality among patients with myocardial infarction presenting without chest pain. JAMA. (2000) 283(24):3223–9. 10.1001/jama.283.24.3223 10866870

[42] Davis LL McCoy TP. An educational and skill-building intervention to improve symptom recognition and interpretation in women with acute coronary syndrome: a pilot study. Dimens Crit Care Nurs. (2019) 38(1):29–37. 10.1097/DCC.0000000000000329 30499790 PMC6278947

[43] Yu H Hong M Zhang M Cao J Chen R Du T. The application of auricular point pressing beans combined with acupoint application in acute coronary syndrome. Chin Med Mod Dist Educ China. (2023) 21(08):133–5. 10.3969/j.issn.1672-2779.2023.08.050

